# Blood-based monitoring identifies acquired and targetable driver *HER2* mutations in endocrine-resistant metastatic breast cancer

**DOI:** 10.1038/s41698-019-0090-5

**Published:** 2019-07-16

**Authors:** Arielle J. Medford, Taronish D. Dubash, Dejan Juric, Laura Spring, Andrzej Niemierko, Neelima Vidula, Jeffrey Peppercorn, Steven Isakoff, Brittany A. Reeves, Joseph A. LiCausi, Benjamin Wesley, Giuliana Malvarosa, Megan Yuen, Ben S. Wittner, Michael S. Lawrence, A. John Iafrate, Leif Ellisen, Beverly Moy, Mehmet Toner, Shyamala Maheswaran, Daniel A. Haber, Aditya Bardia

**Affiliations:** 1 Massachusetts General Hospital Cancer Center, Harvard Medical School, Charlestown, MA 02129 USA; 2 Department of Medicine, Massachusetts General Hospital and Harvard Medical School, Boston, MA 02114 USA; 3 Department of Pathology, Massachusetts General Hospital and Harvard Medical School, Boston, MA 02114 USA; 4 Department of Surgery, Massachusetts General Hospital and Harvard Medical School, Boston, MA 02114 USA; 5 Center for Bioengineering in Medicine, Massachusetts General Hospital and Shriner’s Hospital for Children, Boston, MA 02114 USA; 6 Howard Hughes Medical Institute, Bethesda, MD 20815 USA

**Keywords:** Breast cancer, Cancer genomics

## Abstract

Plasma genotyping identifies potentially actionable mutations at variable mutant allele frequencies, often admixed with multiple subclonal variants, highlighting the need for their clinical and functional validation. We prospectively monitored plasma genotypes in 143 women with endocrine-resistant metastatic breast cancer (MBC), identifying multiple novel mutations including *HER2* mutations (8.4%), albeit at different frequencies highlighting clinical heterogeneity. To evaluate functional significance, we established ex vivo culture from circulating tumor cells (CTCs) from a patient with *HER2*-mutant MBC, which revealed resistance to multiple targeted therapies including endocrine and CDK 4/6 inhibitors, but high sensitivity to neratinib (IC50: 0.018 μM). Immunoblotting analysis of the *HER2*-mutant CTC culture line revealed high levels of HER2 expression at baseline were suppressed by neratinib, which also abrogated downstream signaling, highlighting oncogenic dependency with HER2 mutation. Furthermore, treatment of an index patient with *HER2*-mutant MBC with the irreversible HER2 inhibitor neratinib resulted in significant clinical response, with complete molecular resolution of two distinct clonal *HER2* mutations, with persistence of other passenger subclones, confirming HER2 alteration as a driver mutation. Thus, driver *HER2* mutant alleles that emerge during blood-based monitoring of endocrine-resistant MBC confer novel therapeutic vulnerability, and ex vivo expansion of viable CTCs from the blood circulation may broadly complement plasma-based mutational analysis in MBC.

## Introduction

The treatment of HR+/HER2− breast cancer has markedly evolved with the advent of multiple effective endocrine-based therapies, leading to significantly improved survival in women with metastatic breast cancer (MBC).^1^ Recently demonstrated clinical benefit from combining endocrine therapy with CDK 4/6 inhibitors led to approval of palbociclib (2015), ribociclib (2017), and abemaciclib (2018) as first-line therapies for patients with advanced HR+ breast cancer.^2–4^ However, endocrine resistance ultimately emerges, driven in part by the acquisition of activating mutations in the estrogen receptor (ER) gene *ESR1* mediating resistance to aromatase inhibitors,^5–10^ but other mechanisms governing endocrine resistance are not well understood.

Tumor sampling to identify and monitor the presence of actionable drug resistance-associated mutations is a central tenet of targeted precision oncology. In chronic myeloid leukemia, resequencing of the *BCR-ABL* oncogene may direct use of second line inhibitors,^11^ while in lung cancer, repeat tumor biopsies may identify second site mutations within the driving *EGFR* and *EML4-ALK* oncogenes, or therapeutically actionable alterations in other genes capable of bypassing the inhibited cellular signaling, such as *c-MET* amplification.^12^ On-treatment tumor sampling in HR+ breast cancer, however, has been limited because many patients harbor only bone metastases, which are not readily amenable to biopsy. This together with many resistance-associated mutations in MBC not being druggable result in largely empiric therapeutic regimens for women with metastatic HR+ breast cancer.^1^

Blood-based monitoring is emerging as a robust tool to quantify tumor burden and assess response in patients with solid tumor malignancies, as well as to evaluate the evolution of tumor cell subpopulations, as they decrease or increase in response to therapeutic challenge.^13–15^ Plasma-based genotyping can integrate mutant allele fractions (MAFs) from multiple sites of metastatic disease, identifying distinct clonal derivatives that may have different drug sensitivity and resistance profiles. Indeed, given its ease of application, plasma-based mutation detection has rapidly gained popularity in the clinical setting. However, such plasma-based mutational data alone may not predict drug susceptibility, particularly when multiple mutations are simultaneously present at variable MAFs. In this context, functional validation for identification of driver alterations is critical. The isolation of viable tumor cells (CTCs) from the blood circulation and their expansion in vitro may complement ctDNA mutational analysis, enabling direct testing of drug effects on cell viability and cellular signaling.^5^ Ultimately, correlating clinical response to blood-based parameters is essential to rational treatment decisions. Here, we describe the frequent occurrence of *HER2* mutations, which emerge in patients receiving endocrine-based therapy for HR+ MBC, and utilize patient-derived ex vivo circulating tumor cell (CTC) cultures to demonstrate that such mutations are functional drivers susceptible to novel targeted inhibition.

## Results

### Detection of *HER2* mutations by plasma-based genotyping in HR+/HER2− MBC

We prospectively collected plasma specimens from women with HR+/HER2− MBC who had disease progression on prior endocrine-based regimen, including CDK 4/6 inhibitors (*N* = 143). The blood samples were collected at the time of disease progression, prior to initiation of a second line or subsequent regimen (see Table 1 for detailed clinical characteristics of the cohort, including age at metastatic diagnosis; prior endocrine therapy including aromatase inhibitor, CDK4/6 inhibitors; timing of metastasis; and number of prior therapies). Plasma samples were analyzed by ctDNA genotyping, using a CLIA certified next generation sequencing (NGS)-based clinical assay (Guardant), which includes MAFs for a panel of 73 breast cancer associated genes (Supplementary Table 1).^16^ A total of 14 mutant alleles (11 unique mutations) were identified in 12 of the 143 patients (8.4%). Compared with all patients in the cohort, those with *HER2* mutations had no distinguishing clinical characteristics, including age at diagnosis of metastatic disease, diagnosis as metastatic recurrence versus de novo metastatic disease, presence of visceral versus bone-only metastases, or prior adjuvant treatment including aromatase inhibitors (Table 1).

**Table 1:** Comparison of the clinical characteristics among patients with *HER2*-mutant versus HER2 wild type, metastatic HR+/HER2− breast cancer

Patient characteristics	No. HER2 mutation (*N* = 131)	HER2 mutation (*N* = 12)	*p*-value
Age at primary diagnosis, median (IQR)	51.5 (45.1–61.5)	45.8 (44.3–56.1)	0.34
Age at metastatic diagnosis, median (IQR)	57.3 (49.4–65.9)	58.5 (51.9–66.6)	0.67
Number of patients with de novo metastases (%)	23 (17.6%)	0 (0%)	0.11
Number of prior endocrine therapies for metastatic breast cancer, median (IQR)	1 (0–2)	1 (0–2)	0.64
Number of prior chemotherapies for metastatic breast cancer, median (IQR)	0 (0–2)	0 (0–1)	0.15
Patients with prior CDK 4/6 therapies for metastatic breast cancer (%)	53 (40.5%)	5 (41.7%)	0.93
Patients with prior adjuvant aromatase inhibitors for localized breast cancer (%)	49 (37.4%)	7 (58.3%)	0.16

**Fig. 1:**
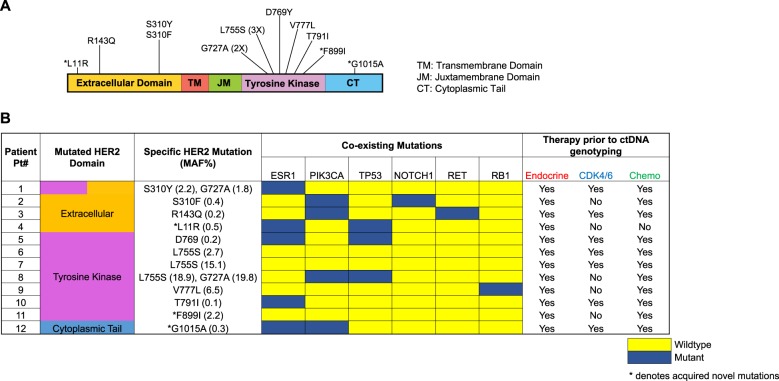
HER2 and coexisting mutations in patients with HR+ metastatic breast cancer. **a** Graphic representation of the positions of somatic *HER2* mutations identified using ctDNA analysis of patients enrolled in this study. Asterisk indicates novel *HER2* mutations. **b** List of gene mutations coexisting with the somatic *HER2* mutations. The mutant allelic frequencies (MAF) of each patient and the therapies received prior to ctDNA analysis are shown. Asterisk indicates novel *HER2* mutations

Of the eleven unique HER*2* mutations, eight (72.8%) have been previously reported in cancer, while three (27.2%) are novel variants: L11R, F889I, and G1015A. While the functional significance of these novel variants is uncertain, we note that they are located within evolutionary conserved residues: F889I and G1015A affect amino acids that are conserved in all four species analyzed (human, pig, mouse, and rat), while L11R is conserved in human and pig. All together, the *HER2* mutations span multiple domains, as depicted in Fig. 1a. Of the eleven unique mutations, most are in the tyrosine kinase domain (*N* = 6 unique mutations, accounting for nine mutant alleles), while others are in the extracellular domain (*N* = 4) and cytoplasmic tail (*N* = 1), consistent with different mechanisms implicated in the activation of this ligand-independent receptor. Most importantly, for eight cases, matched primary tumor specimens were available. However, the *HER2* mutations were detectable in none of these, suggesting that they likely emerged under therapeutic pressure during the course of endocrine-based therapy (Table 2).

**Table 2:** Detailed description of *HER2* mutations, coexisting mutations, mutant allele frequencies (MAF), and prior treatment history in patients with *HER2-*mutant metastatic HR+/HER2− breast cancer

Pt	HER2 mutation (MAF %)	HER2 mutation reported/novel	HER2 mutation in tissue	Therapy prior to ctDNA testing			Adjuvant versus metastatic endocrine therapy	Time between tissue and ctDNA	Mutations in tissue and ctDNA	Biopsy site	Coexisting ctDNA mutations (MAF%)
				Endocrine	CDK 4/6	Chemo					
1	S310Y (1.3)	Reported	No	Yes	Yes	Yes	Adjuvant and metastatic	4 months	None	Liver	ESR1 D538G (2.7), ESR1 E380Q (0.8), EGFR V536M (2.1), NF1 Y489C (0.5)
	G727A (3.9)	Reported									
2	S310F (0.4)	Reported	No	Yes	No	Yes	Adjuvant and metastatic	23 months	ALK, APC, CDH1, PIK3CA	Lymph node	ALK R1084C (0.2), APC S331L (0.2), ARID1A G838A (0.9), ARID1A E1006K (0.7), ARID1A S662T (0.7), ARID1A D1963N (0.3), ARID1A Q790Q (0.3), BRCA1 E1033Q (1.9), BRCA1 E761K (0.3), BRCA2 Q684K (0.7), BRCA2 Q1138Q (0.2), CDH1 V391I (0.4), NF1 E1790K (0.1), NOTCH1 E1567K (0.1), PIK3CA E545K (0.3), TSC1 E1106K (0.3)
3	R143Q (0.2)	Reported	No	Yes	Yes	Yes	Adjuvant and metastatic	2 months	None	Breast	ALK R1347Q (0.2), PIK3CA E542K (2.0), RET M918T (2.2)
4	L11R (0.5)	Novel	N/A	Yes	No	No	Adjuvant and metastatic	N/A	N/A	N/A	AKT1 E17K (21.3), ARAF T213P (0.3), ESR1 D538G (11.3), FGFR3 K404N (0.6), SMAD4 Q442* (7.5), SMAD4 Q180* (2.1), SMAD4 S242* (0.2), SMAD Q183* (1.5), TP53 R248Q (0.1)
5	D769Y (0.2)	Reported	No	Yes	Yes	Yes	Adjuvant and metastatic	23 months	None	Lymph node	ESR1 Y537C (0.3), RB1 N522fs (1.1), TP53 F270fs (51.4)
6	L755S (2.7)	Reported	No	Yes	Yes	Yes	Adjuvant	22 months	None	Unspecified	None
7	L755S (15.1)	Reported	No	Yes	Yes	Yes	Adjuvant and metastatic	27 months	None	Femur	AR P283A (3.8)
8	L755S (18.9)	Reported	N/A	Yes	No	Yes	Metastatic	N/A	N/A	N/A	PIK3CA (54.0), TP53 K164*
	G727A (19.8)	Reported									
9	V777L (6.5)	Reported	N/A	Yes	No	Yes	Adjuvant	N/A	N/A	N/A	RB1 E19fs (0.4)
10	T791I (0.1)	Reported	N/A	Yes	Yes	Yes	Adjuvant and metastatic	N/A	N/A	N/A	ARID1A T493S (0.2), ESR1 E380Q (1.4), FGFR1 S353C (0.4)
11	F899I (2.2)	Novel	No	Yes	No	Yes	Adjuvant and metastatic	5 months	AKT1	Skin	ATM R3008H (0.2)
12	G1015A (0.3)	Novel	No	Yes	Yes	Yes	Adjuvant and metastatic	87 months	PIK3CA	Breast	AIRID1A R1287T (0.2), CCNE1 M234I (1.1), EGFR E866Q (0.2), ESR1 Y537N (0.4), NF1 H1528D (0.2), PIK3CA E722D (0.3), PIK3CA H1047R,(59) SMAD4 K428* (34.9), SMAD4 R445* (1.0)

*indicates premature termination codon

Two patients, Patient 1 and Patient 8, had two distinct *HER2* mutations simultaneously detected in ctDNA, suggesting the polyclonal nature of these mutations in patients with MBC (Fig. 1b). MAFs for all *HER2* mutations ranged from 0.1% to 19.8% (Table 2). Besides the *HER2* mutations, multiple coexisting mutations were detected, albeit at different MAFs, highlighting clonal heterogeneity. Common coexisting mutations included *ESR1* (41.6%), *TP53* (25%), and *PIK3CA* (33.3%), all of which have been reported in endocrine resistant breast cancer.^17^ Interestingly, half the *PIK3CA* mutations were also present in the patients’ pretreatment metastatic tumor biopsies, which suggests these were possibly pre-existing truncal mutations (i.e. dominant mutations shared by subsequent clones). The pre-existing PIK3CA mutations were H1047R and E545K, both known hotspot mutations, and in subsequent ctDNA sampling the former remained the dominant mutation (59%), and the latter in a percentage comparable to the accompanying mutations (0.3%). One patient (12, Table 2) also appeared to have acquired a small percentage PIK3CA mutation E722D, though the quantity was comparatively low (0.3%), and this is not a known hotspot mutation. In contrast, the activating *ESR1* mutations, like the *HER2* mutations, were detected only in the posttreatment ctDNA sample, indicating acquired selection during drug therapy. The *TP53* mutations in ctDNA were also not detected in the tissue biopsy, although there is incomplete overlap in the specific *TP53* mutations detected by the tumor versus ctDNA assays. Of note, within individual patients, *HER2* and *ESR1* subclonal mutations appeared at different allele frequencies, raising the possibility that these two distinct resistance mechanisms contribute toward the full complement of drug resistance in heterogeneous tumor populations (Table 2).

### Analysis of patient-derived ex vivo cultured CTCs with *HER2* mutation

To provide functional validation for the *HER2* mutations detected by plasma genotyping, we analyzed viable CTCs that were successfully expanded in vitro from a subset of patients in the cohort. We used a microfluidic “negative depletion” CTC-iChip platform^5,18,19^ to process 10 ml of whole blood, effectively removing normal hematopoietic cells and enriching for unmanipulated viable CTCs. CTCs were successfully cultured from Patient 2 (Fig. 1a) who had a *HER2* S310F mutation identified by ctDNA genotyping, and the *HER2* mutation was confirmed in the CTC-derived cultures.

**Fig. 2:**
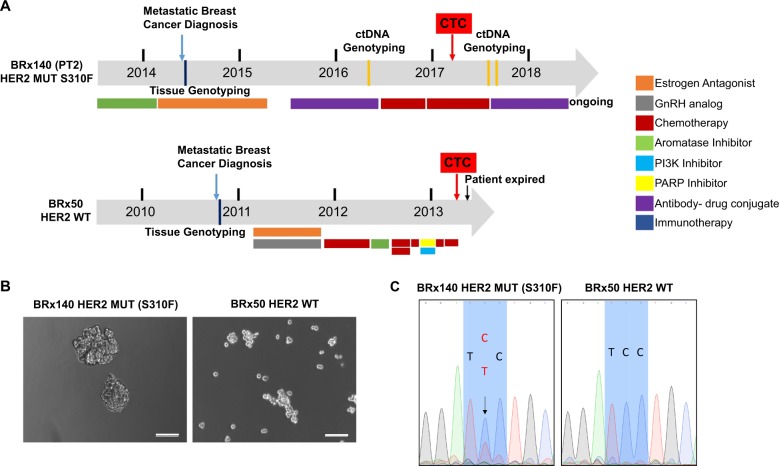
Ex vivo culture of CTCs from two patients with hormone receptor positive metastatic breast cancer. **a** Summary of the treatment history of patients with metastatic breast cancer whose CTCs were cultured ex vivo. BRx140 harbors mutant (Mut) *HER2* and wild-type ESR1 and BRx50 harbors wild-type (WT) HER2 and *ESR1* mutant alleles. Clinical status of patients BRx50 and BRx140 is listed above the time line bar and treatment regimens are listed below the bar. The time of CTC isolation followed by successful expansion ex vivo is shown as red boxes on the time line bar. **b** Bright field images showing ex vivo cultures of CTCs from patients BRx140 and BRx50. Scale bar represents 100 μm. **c** Sanger sequencing of the HER2 allele showing the presence of heterozygous *HER2* (S310F) mutation in BRx140 and wild-type HER2 in BRx50. Nucleotide base change indicated with a black arrow

The clinical therapeutic history and timing of CTCs cultured from Patient 2 (Fig. 1a; BRx140) are shown in Fig. 2a, along with a comparable CTC culture from a patient (BRx50) with wild-type *HER2*.^5^ The corresponding CTC cultures are illustrated in Fig. 2b. A heterozygous *HER2* mutation (S310F) is evident in BRx140 cells, identical to the mutation identified in the ctDNA from Patient 2 (Fig. 2c). This mutation was not detected in a tumor infiltrated lymph node resected prior to the development of endocrine drug resistance, consistent with an acquired genetic event. Patient characteristics and additional mutations scored in *HER2*-mutant CTC cell line (BRx140) and HER2-wild-type CTC cell line (BRx50) are described in Supplementary Table 1A and B. The patient-derived CTC cell line BRx140 provided a robust tool for us to study functional properties of mutant *HER2*, compared to the BRx50 CTCs, which are wild type for *HER2*, but harbor an *ESR1* mutation^5^ (Supplementary Fig. 1A).

**Fig. 3:**
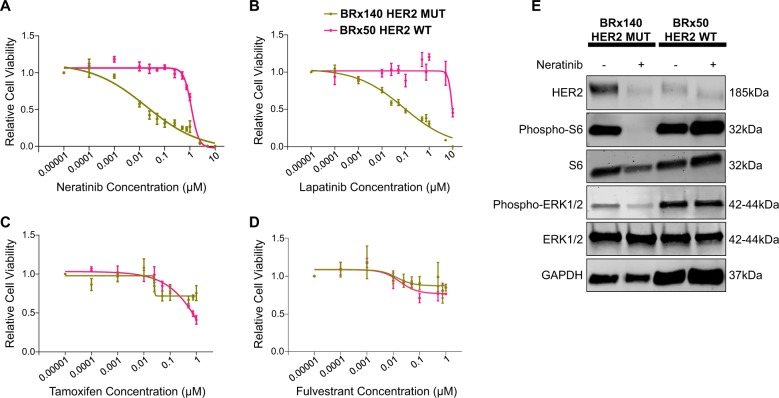
Impact of various targeted therapies on growth of the patient derived *HER2*-mutant CTC cell line, compared to HER2-wild-type CTC line. **a**–**d** BRx140 and BRx50 were treated with increasing concentrations of neratinib (**a**), lapatinib (**b**), selective estrogen receptor modulator, tamoxifen (**c**), and selective estrogen receptor degrader, fulvestrant (**d**), for 5 days. Dose response curves show that the *HER2*-mutant CTC line, BRx140, is highly sensitive to neratinib treatment and moderately sensitive to lapatinib treatment, compared to the HER2-wild-type CTC line BRx50. Both CTC lines are resistant to tamoxifen and fulvestrant. **e** Neratinib treatment inhibits HER2 signaling in the *HER2*-mutant CTC cell lines. The CTC cell lines shown were treated with 100 nM neratinib for 24 h and proteins were analyzed for HER2, phopho-S6, and phosho-ERK1/2 expression. Total S6 and ERK1/2 are shown. GAPDH was analyzed as control for equal loading of proteins. All blots and gels are accompanied by the locations of molecular weight/size markers

Treatment of the cultured CTCs with the ER degrader fulvestrant, the ER modulator tamoxifen, or the CDK4/6 inhibitors, palbociclib or ribociclib, showed no difference between cells harboring or lacking a *HER2* mutation (Supplementary Fig. 1B). The relative insensitivity of these cultured CTCs to these agents is consistent with the cases of hormone-refractory breast cancers from which they were derived (Supplementary Fig. 1B). In contrast, *HER2*-mutant CTCs (BRx140) were highly sensitive to treatment with the HER2 inhibitor neratinib (IC50: 0.018 μM) and moderately sensitive to lapatinib treatment (IC50: 0.0837 μM), whereas CTCs with wild-type *HER2* (BRx50) were resistant to both neratinib (IC50: 1.151 μM) and lapatinib (IC50: 9.603 μM) (Fig. 3a–d).

Immunoblotting analysis of the *HER2*-mutant BRx140 CTCs showed high levels of HER2 expression at baseline, which was suppressed by neratinib exposure, consistent with its known effect on receptor internalization and degradation.^20^ No such effect was seen in the BRx50 cells with wild-type *HER2* expression. Remarkably, phosphorylation of the downstream signaling effectors S6 and ERK was abrogated following treatment of *HER2*-mutant CTCs with neratinib (Fig. 3e), indicating that signaling by mutant *HER2* is the primary driver of proliferative signals in these cells. In contrast, phospho-S6 and phospho-ERK levels in the *HER2*-wild-type CTCs (BRx50) were unaffected by neratinib, consistent with the presence of alternative drivers of cellular proliferation. Thus, functional studies of HER2 targeting in CTC-derived cultures from a patient who had acquired a *HER2* mutation during the course of endocrine therapy suggests that the emergence of this mutation may drive a new oncogenic dependency that is susceptible to therapeutic intervention.

### Therapeutic response in a patient with ctDNA positive for two *HER2* mutations

To evaluate therapeutic inhibition, we evaluated the treatment patterns. In this cohort, three patients with *HER2* mutant alleles identified by ctDNA genotyping were treated with neratinib, an irreversible, pan-HER kinase inhibitor. One patient (index Patient 1 in Fig. 1b) had two coexisting *HER2* mutations (S310Y and G727A) and two activating *ESR1* mutations (D538G and E380Q) based on plasma genotyping and had adequate follow-up for determination of treatment efficacy. Of the two other patients with HER2 mutant alleles who received neratinib, one had rapid disease progression before receiving neratinib (off-label compassionate use) and could only receive a few doses before discontinuation of all medical therapies and enrollment in hospice care, and the other recently started treatment and has not yet received the required follow-up for evaluation of treatment efficacy. The other nine patients received different therapies, consistent with the availability of multiple treatment options for MBC (data-cut off September 30, 2018).

**Fig. 4:**
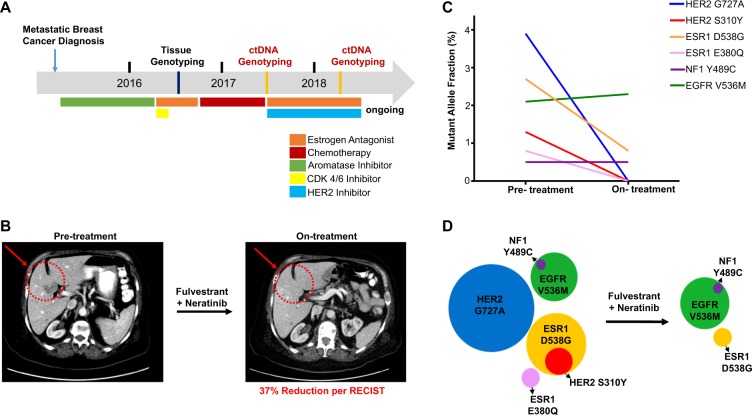
Clinical and molecular responses in a *HER2*-mutant index patient (Patient 1) receiving fulvestrant and neratinib. **a** Summary of the treatment history of Patient 1. Clinical status of the patient is listed above the timeline bar and treatment regimens are listed below the bar. **b** Restaging CT scan obtained before and during treatment with fulvestrant and neratinib of a *HER2-*mutant index patient demonstrates significant tumor reduction (37% reduction per RECIST at 10 months) consistent with objective partial response. Target lesions in the liver are indicated with a circle and red arrow. **c** Changes in the MAFs of mutations in the pretreatment and on-treatment plasma specimens obtained from the *HER2*-mutant index patient receiving fulvestrant and neratinib demonstrate the disappearance of *HER2* mutant clones, partial decreases in *ESR1*-mutant clones, and no major change in the *NF1-* and *EGFR-*mutant containing subclones. **d** Proposed reconstruction of pretreatment and posttreatment genetic clones and subclones in Patient 1 demonstrating resistance and sensitivity to fulvestrant and neratinib treatment. The diameter of each circle represents the allelic frequency of the relevant mutation. The subclones containing both *NF1* and *EGFR* mutations as well as *ESR1* and *HER2* mutations were derived based on the parallel changes in the MAFs during treatment with neratinib and fulvestrant

The index patient is noteworthy for having received multiple prior endocrine therapies, including aromatase inhibitor and SERD, for HR+/HER2− MBC (Fig. 4a). She was treated with neratinib and fulvestrant, as part of an IRB-approved basket clinical trial, resulting in a significant reduction in tumor volume (37% reduction per RECIST at 10 months; Fig. 4b). Repeat ctDNA analysis using the same assay after 6 months of treatment showed a complete molecular response, with disappearance of both *HER2* mutations. Interestingly, while the *HER2* mutant alleles targeted by neratinib rapidly resolved, the pharmacodynamic effect of fulvestrant on ESR1 mutant alleles appeared to be more modest. The E380Q *ESR1* mutations, which had a very low MAF, resolved, but the more abundant D538G *ESR1* mutation was only partially reduced. The MAF of other mutations, including *NF1* and *EGFR* mutations, was unaffected by the neratinib and fulvestrant combination therapy (Fig. 4c).

The absolute level of MAF for different mutations and their rate of change as a function of therapy allowed modeling of tumor subpopulations in this patient, suggesting four dominant clones: a major tumor cell population harboring *HER2* G727A, which declined rapidly on-treatment; a second population harboring *ESR1* D538G, which declined slowly, but within which a smaller fraction with *HER2* S310Y disappeared rapidly on-treatment; a third subpopulation with *EGFR* V536M, a fraction of which also had *NF1* Y489C, which remained stable on-treatment; and a fourth minor subpopulation with a *ESR1* mutation E380Q, which became undetectable on-treatment (Fig. 4d). Thus, three of the four identified tumor subpopulations harbored four different resistance alleles, with the *HER2* mutations displaying prompt response to neratinib, while the *ESR1* mutations appeared to decline more modestly following fulvestrant. The total ctDNA tumor burden was reduced (total MAF from 3.9% to 2.3%), consistent with the presence of a subset of tumor cells harboring *HER2* mutations and reflecting the partial clinical response observed in the patient.

## Discussion

In this translational study, we demonstrate the emergence of *HER2* mutations, detectable by plasma DNA sequencing, in women with HR+/HER2− MBC who have developed clinical resistance to endocrine-based combination therapies, and provide functional validation based on patient-derived CTC culture models. Acquired mutations in the setting of clinical progression often arise along with mutations in other genes, and may be present as dominant clones, or less prevalent subclones. It is in this context that functional studies are required to demonstrate the physiological significance of these acquired mutations as “drivers” of proliferation, rather than incidental passengers. We provide two lines of evidence to support the clinical importance of the *HER2* mutations: first, in one patient treated with the HER2 inhibitor neratinib who was evaluable for response, the two subclonal *HER2* mutations disappeared within 6 months of treatment initiation, whereas other concurrent mutations, marking other *HER2*-wild-type subclones, were not changed. The patient remained in prolonged partial remission for >1 year. Second, using ex vivo cultured CTCs from a patient with an acquired *HER2* mutation, we demonstrate that, in these but not in *HER2*-wild-type CTCs, neratinib abrogates the major downstream signaling pathways for cell survival and proliferation, an effect that is associated with exquisite sensitivity of the cultured CTCs to HER2 inhibition. Together, these observations point to the emergence of *HER2* mutations as a recurrent mechanism of disease progression in HR+, HER2− breast cancer, and suggest that this mechanism of clinical resistance to endocrine therapy may be associated with de novo sensitivity to HER2 inhibition in some patients.

Activating mutations in *HER2* have been reported in small subsets of multiple different cancers.^21–24^ In primary breast cancer, they are particularly rare, with an estimated prevalence of 1.8% in primary tumors based on The Cancer Genome Atlas analysis.^25^ While this manuscript was in preparation, another group reported a high prevalence of *HER2* mutations (6%) based on tissue genotyping of metastatic specimens in endocrine-resistant HR+/HER2− breast cancer,^26^ and two groups demonstrated enrichment of *HER2* mutations in ctDNA after treatment with endocrine-based therapy.^27,28^ As in these studies, we cannot determine whether these mutations were present below detection at the time of diagnosis and were subsequently enriched during the course of endocrine therapy, or whether they were completely absent from the original tumor but arose through de novo mutation during cancer progression. In our study, none of the eight ctDNA-positive patients for whom primary tumor material was available for sequencing had evidence of detectable *HER2* mutant alleles within the archival tissue specimen (<0.1% MAF). Interestingly, none of the *HER2* mutant cases had identifiable metastatic disease at presentation, thus it is unlikely these mutations arose within a specific metastatic tumor lesion that had not received prior endocrine-based therapy. Future studies would benefit from collecting plasma ctDNA at the time of diagnosis to confirm the presence or absence of mutations. In addition, detailed information about certain clinical characteristics, such as disease-free interval (from early stage to metastatic) and histology (lobular versus ductal), was not available in the database and the study observations require validation in additional studies.

Unlike the prototypical *HER2* gene amplifications that define a major subset of primary breast cancer and are highly correlated with response to HER2-targeting antibodies, point mutations in *HER2* are less well characterized, and their occurrence within multiple domains of the receptor, including extracellular, catalytic, and cytoplasmic tail, make them less readily interpretable than recurrent kinase domain mutations, such as observed in the related receptor EGFR.^29^

Moreover, unlike HER2-amplified breast cancer, tumors harboring *HER2* mutations appear to be resistant to standard HER2-targeted antibodies, including trastuzumab.^23^ *HER2*-mutant breast cancers exhibit mixed responses to the most commonly used small molecular inhibitor, lapatinib, although they may be more sensitive to more potent irreversible HER2 inhibitors, such as neratinib.^23,30^ Lapatinib and neratinib were chosen to test the response to reversible and irreversible HER2 inhibition, respectively, utilizing two drugs that are FDA approved. The clinical efficacy of neratinib in metastatic *HER2* mutant solid tumors, including breast cancer, was recently described in a landmark “basket” trial.^31^ In the study by Hyman et al., an objective response rate of 32% (95% CI: 15–54%) was noted in patients with *HER2*-mutant breast cancer (*N* = 25), with responsive tumors having mutations in extracellular domains as well as the tyrosine kinase domain. Variable clinical responses were also reported with neratinib in a trial of *HER2*-mutant MBC, screened by tissue-based genotyping.^32^ Together with our own study, these results extend the therapeutic application of HER2 targeted therapies to *HER2* mutations that emerge after endocrine-based therapy and are identified at various allele frequencies coexisting with other mutations. The prevalence of *HER2* mutations appears to be considerably higher in these patients, who received endocrine therapies with or without CDK4/6 inhibitors, compared with the *HER2* mutation frequency established in untreated primary breast cancer.

Oncogene addiction by cancer cells was classically defined as resulting from their inherent wiring through a dominant or “truncal” mutation that drives oncogenic signaling in the cell.^33^ As such, it is uncertain whether mutations acquired during the course of cancer progression may mediate similar dependencies, particularly if they constitute subclonal late somatic events that arise in the context of multiple other mutations. Indeed, most *HER2* mutations have been functionally annotated using ectopic expression in preclinical model, which cannot predict their oncogenic dependence when arising endogenously in cancer cells.^34^ The difficulty in assessing the clinical relevance of acquired *HER2* mutations is all the more critical with the advent of liquid biopsies, which may identify large numbers of potentially actionable mutations present at low allele frequencies. For example, besides HER2 mutations we also observed ARID1A mutations that suggests emergence of epigenetic escape or adaptive response mechanisms, requiring further evaluation in additional studies. While larger data sets will be required to confirm our observations, the data presented here suggests that even at subclonal allele frequencies, the acquisition of *HER2* mutations denotes a potential therapeutic opportunity. These data are supported by recently published research identifying activating mutations in HR+ breast cancer, which similarly appears to confer resistance to hormonal therapy, but sensitivity to neratinib.^35^ Similarly, this study describes mutations found in a relatively small population, eight patients, thus our work compliments these findings and supports the principle of acquired activating mutations conferring both endocrine resistance, as well as a new sensitivity to HER2-targeted therapy. As such, *HER2* mutations join *ESR1* mutations as recurrent mechanisms of acquired resistance to endocrine combination therapy, which may coexist within individual patients, and both of which have potentially important therapeutic implications.

Plasma ctDNA analysis has previously been used to track the emergence of activating mutations in *ESR1*, which are common in women treated with aromatase inhibitors.^10^ The distinct tracking of *HER2* and *ESR1* ctDNA mutations in our index patient responding to combined neratinib and fulvestrant therapy is consistent with the presence of multiple coexisting tumor subpopulations, each responding with unique dynamics. The diverse clonal nature of endocrine drug resistance is exemplified by patient-derived cultured CTCs exhibiting one or the other mechanism. Treatment options in patients who harbor multiple subclonal mechanisms of endocrine drug resistance are not established and may require ongoing assessment of dominant clones following treatment interventions. We note, however, that the sustained response in our index patient is correlated with the dramatic decline in *HER2* MAF, with a more modest response to ER targeting fulvestrant, raising the possibility that HER2-activated tumor clones may contribute disproportionately to tumor proliferation and expansion in this patient.

In summary, the emerging use of ctDNA genotyping to monitor response and potentially guide therapeutic choices brings with it the challenge of evaluating the predictive and functional value of multiple subclonal mutant alleles as potentially therapeutic targets. The fact that acquired oncogenic driver mutations may mediate resistance to an initial therapeutic regimen, while establishing sensitivity to a second treatment option highlights new opportunities in precision targeting of acquired mutations to prolong clinical responses for patients with MBC.

## Methods

### Patient selection and genomic analysis

Blood specimens were collected after informed consent as either standard of care for clinical genotyping or under an IRB approved tissue/blood collection protocol from patients with histologically confirmed HR+/HER2− MBC at the Massachusetts General Hospital and underwent ctDNA testing utilizing a CLIA certified ctDNA assay (Guardant), an NGS-based commercially available assay that detects ctDNA down to 0.1% allelic fraction with a clinical sensitivity of 85% (compared to 80.7% in tissue) and 99.8% specificity.^16^ We also reviewed the genomic profile results of the matched patient-derived primary or metastatic tumor biopsies, and the CTC cultures utilizing the SNaPshot-NGS clinical assay, an institutional anchored multiplex PCR assay that detects SNVs and indels in tissue biopsies. Independent chart review was utilized to gather data on clinico-pathological characteristics, mutation data (from Guardant clinical reports and Snapshot clinical reports), and clinical outcomes.

### Statistical analysis

Statistical significance was defined as *P* < 0.05. Stata (StataCorp. 2015. Stata Statistical Software: Release 14. College Station, TX: StataCorp LP) was used to perform analyses.

### Enrichment of patient CTCs

Informed consent (written) was obtained from MBC patients for CTC collection as per Dana Farber/Harvard Cancer Center institutional review board approved protocol (DF/HCC 05–300). Twenty millilitres of whole blood was obtained from BRx140 in two EDTA tubes and CTCs were isolated using the microfluidic CTC-iChip as previously described.^19^ In brief, whole blood was incubated with biotinylated antibodies against CD45 (clone 2D1; R&D Systems), CD66b (clone 80H3; AbD Serotec), and CD16 (Janssen Diagnostics) followed by incubation with Dynabeads MyOne Streptavidin T1 (Invitrogen) to achieve magnetic labeling of leukocytes. This blood was then processed through the CTC-iChip and the product was collected in CTC culture media (see below) under aseptic conditions for derivation of patient-specific CTC cultures.

### Ex vivo CTC culture

CTC cultures were grown in CTC media comprising of RPMI-1640, bFGF (20 ng/ml), EGF (20 ng/ml), 1X B27, and 1X antibiotic/antimycotic (Life Technologies) in ultralow attachment flasks (Corning) at 37 °C under hypoxic conditions (4% O_2_) with 5% CO_2_. Cultures were routinely checked for mycoplasma with the MycoAlert and Lonza Kit and tested for authentication via STR profiling by Genetica DNA Laboratories (a LabCorp brand; Burlington, NC) using the commercially available PowerPlex® 16HS amplification kit (Promega Corporation; mouse marker included) and GeneMapper ID v3.2.1 software (Applied Biosystems). The CTC line BRx 50 has been previously described.^5^

### Immunoblot

A total of 100 nM of Neratinib was added to 5 × 10^5^ cells for 24 h and 10 μg each of protein lysates were separated on SDS/4–15% polyacrylamide gels (Bio-Rad) and transferred onto nitrocellulose membranes (Invitrogen). The blots were incubated with antibodies directed against GAPDH [(14C10) cell signaling #2218], HER2 [(29D8) cell signaling #2165], Erk1/2 [cell signaling #9102], Phospho-Erk1/2 [(Thr202/Tyr204) cell signaling #9101], phospho-S6 ribosomal protein [(Ser235/236) cell signaling #2211], and S6 ribosomal protein [(5G10) cell signaling #2217] and with the relevant secondary antibodies and visualized using Odyssey Clx (LI-COR). All blots were derived from the same experiment and were processed in parallel. In Supplementary Material, all blots and gels are accompanied by the locations of molecular weight/size markers and uncropped scans (Supplementary Fig. 2).

### Dose response curves for drug sensitivities

A total of 1000 cells were seeded in a 96-well ultra-low attachment plate (Corning). Increasing concentrations of neratinib (HKI-272) (Selleckchem S2150), lapatinib (Selleckchem S2111), tamoxifen (Selleckchem S1238), and fulvestrant (Selleckchem S1191) were added to quadruplicate samples. The cells were incubated with the above drugs for 5 days under hypoxic conditions. Viability was measured using CellTiter-Glo Luminescent Cell Viability Assay per the manufacturer’s instructions.

### CDK4/6 inhibitor treatment

A total of 2000 cells were seeded in a 96-well ultralow attachment plate (Corning). A total of 500 nM ribociclib (LEE011) (Selleckchem S7440) and 500 nM palbociclib (PD-0332991) HCl (Selleckchem S1116) were added to quadruplicate samples for 3 days and viability was measured using CellTiter-Glo Luminescent Cell Viability Assay per the manufacturer’s instructions.

### DNA extraction, PCR, and sanger sequencing

DNA was extracted using All Prep DNA/RNA Mini Kit from Qiagen per the manufacturer’s instruction. PCR was performed with 100 ng of DNA and primers flanking the mutation using KAPA HiFi HotStart ReadyMix PCR Kit based on the manufacturer’s instructions. PCR products were purified using QIAquick PCR Purification Kit from Qiagen followed by Sanger sequencing by Eton Bioscience Inc.

ESR1 forward primer: 5′-TGGAAGTCACCTGCATAGCAAATACCCTG-3′

ESR1 reverse primer: 5′-GCAAATGAATGGCCACTCATCTAGAAAGCC-3′

HER2 forward primer: 5′-TAACAGATCACCTATTTACTGATGGGC-3′

HER2 reverse primer: 5′-CACTGACAGGGGATATAGGGACACTTGTA-3′

## Supplementary information

Supplementary Information

## Data Availability

Sequencing data submitted to GenBankBank, accession code: MN047439, MN047440, MN047441, and MN047442. Additional data available on request from the authors.

## Supplementary information

**Supplementary Information** accompanies the paper on the *npj Precision Oncology* website (10.1038/s41698-019-0090-5).
