# Supplementary material for: Blood-based monitoring identifies acquired and targetable driver HER2 mutations in endocrine-resistant metastatic breast cancer
Source: NPJ Precis Oncol. 2019 Jul 16;3:18. doi: 10.1038/s41698-019-0090-5 (PMC6635494; doi:10.1038/s41698-019-0090-5)
Supplement: Supplementary file 1 — Supplementary Information [file 41698_2019_90_MOESM1_ESM.ppt]

## Slide 1
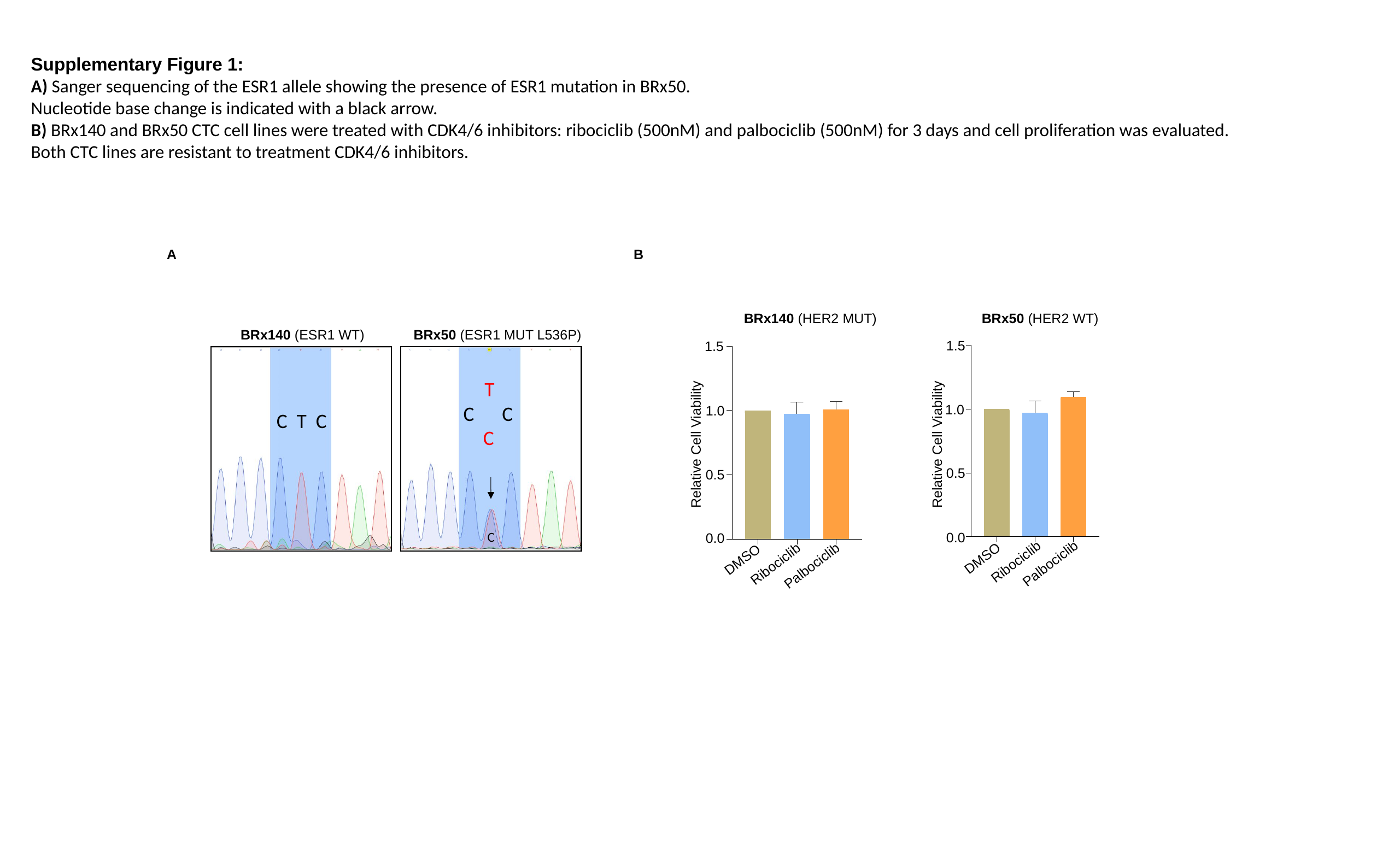

Supplementary Figure 1:
A) Sanger sequencing of the ESR1 allele showing the presence of ESR1 mutation in BRx50.
Nucleotide base change is indicated with a black arrow.
B) BRx140 and BRx50 CTC cell lines were treated with CDK4/6 inhibitors: ribociclib (500nM) and palbociclib (500nM) for 3 days and cell proliferation was evaluated.
Both CTC lines are resistant to treatment CDK4/6 inhibitors.
A
B
BRx140 (HER2 MUT)
BRx50 (HER2 WT)
1.5
1.5
1.0
1.0
Relative Cell Viability
Relative Cell Viability
0.5
0.5
0.0
0.0
DMSO
DMSO
Ribociclib
Palbociclib
Ribociclib
Palbociclib
BRx140 (ESR1 WT)
BRx50 (ESR1 MUT L536P)
T
C C
C T C
C
C

## Slide 2
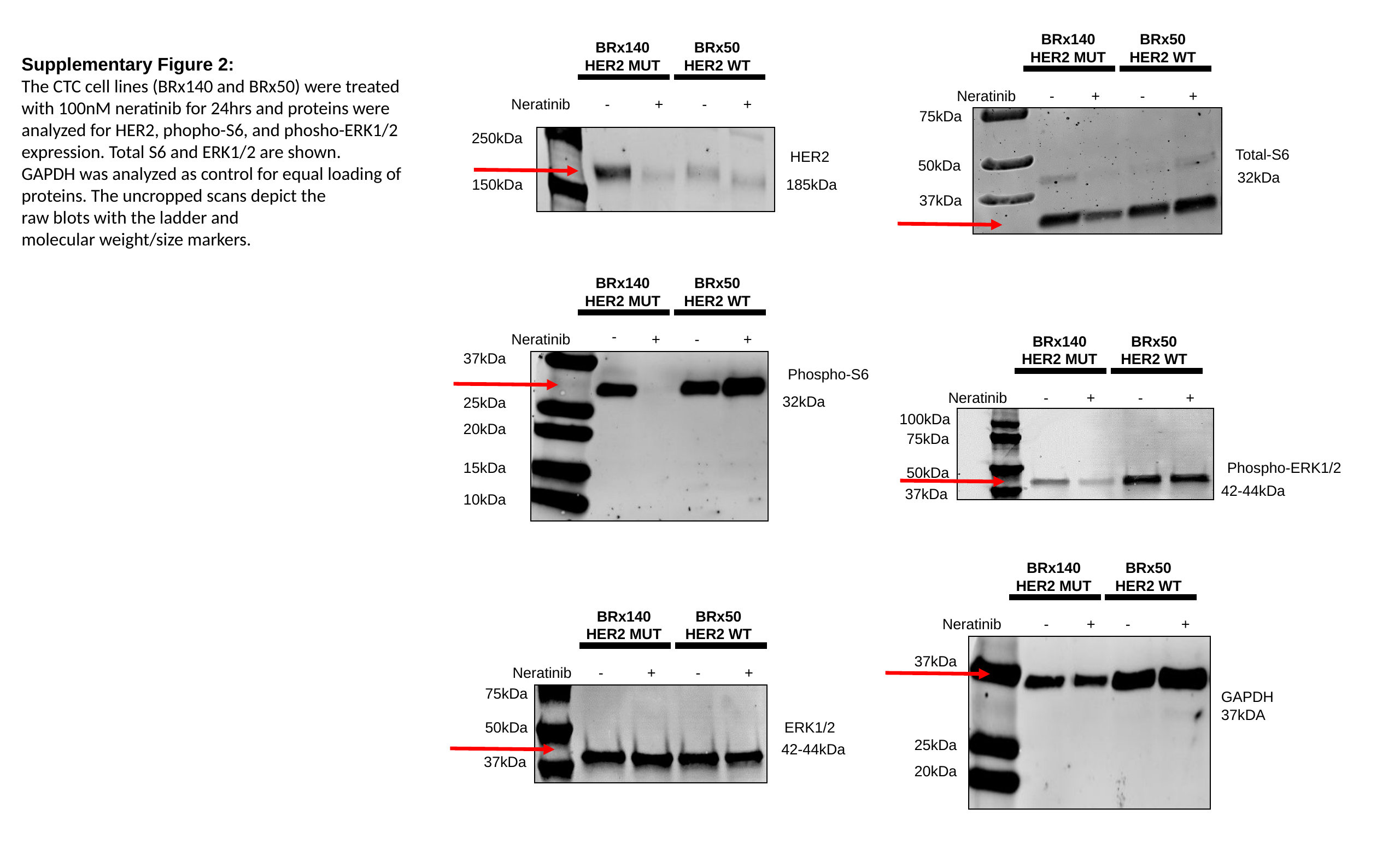

BRx140
HER2 MUT
BRx50
HER2 WT
Neratinib
-
+
-
+
75kDa
Total-S6
50kDa
32kDa
37kDa
BRx140
HER2 MUT
BRx50
HER2 WT
Neratinib
-
+
-
+
250kDa
HER2
150kDa
Supplementary Figure 2:
The CTC cell lines (BRx140 and BRx50) were treated
with 100nM neratinib for 24hrs and proteins were
analyzed for HER2, phopho-S6, and phosho-ERK1/2
expression. Total S6 and ERK1/2 are shown.
GAPDH was analyzed as control for equal loading of
proteins. The uncropped scans depict the
raw blots with the ladder and
molecular weight/size markers.
185kDa
BRx140
HER2 MUT
BRx50
HER2 WT
-
Neratinib
+
-
+
37kDa
Phospho-S6
32kDa
25kDa
20kDa
15kDa
10kDa
BRx140
HER2 MUT
BRx50
HER2 WT
Neratinib
-
+
-
+
100kDa
75kDa
Phospho-ERK1/2
50kDa
42-44kDa
37kDa
BRx140
HER2 MUT
BRx50
HER2 WT
Neratinib
-
+
-
+
37kDa
GAPDH
37kDA
25kDa
20kDa
BRx140
HER2 MUT
BRx50
HER2 WT
Neratinib
-
+
-
+
75kDa
50kDa
ERK1/2
42-44kDa
37kDa

## Slide 3
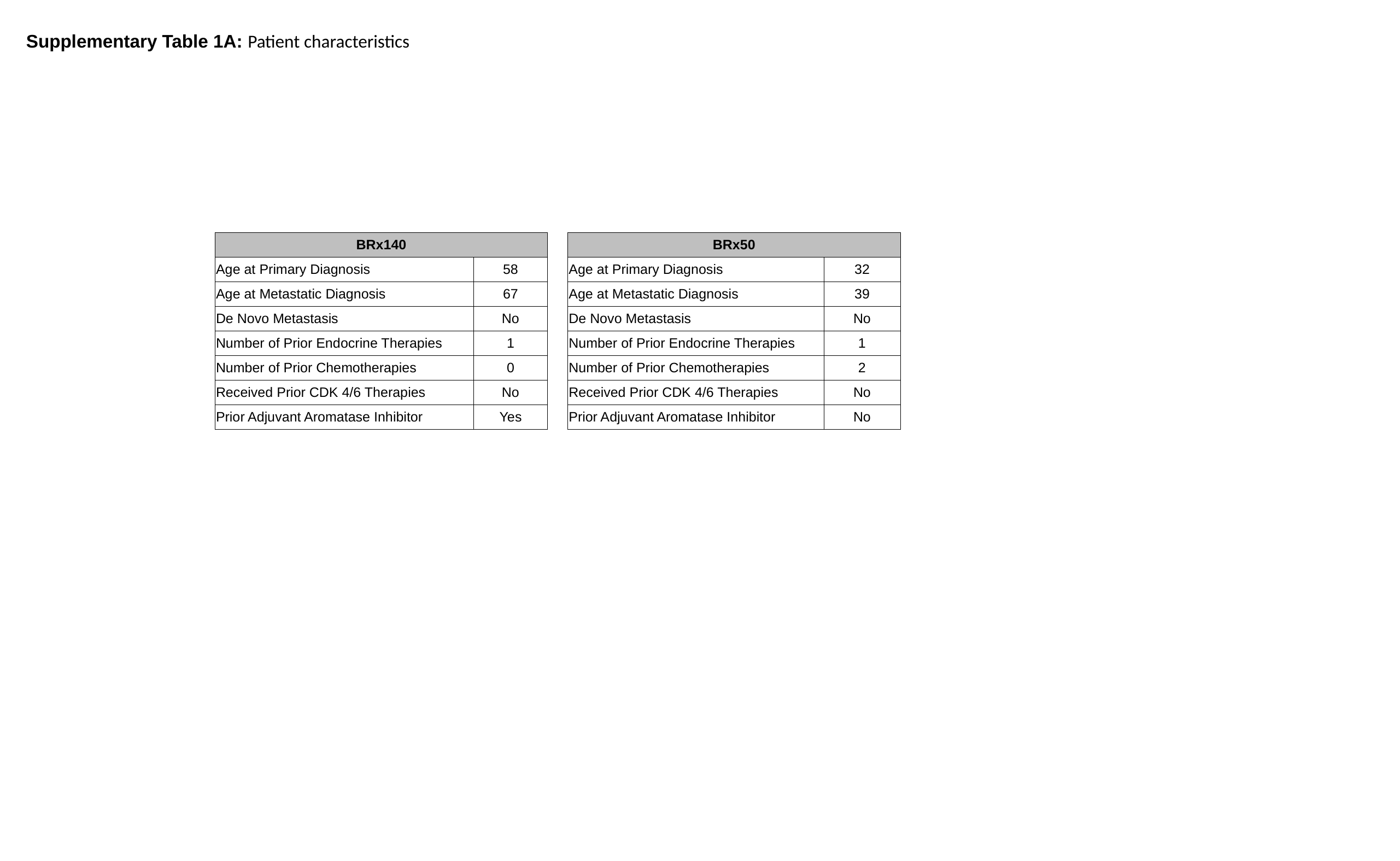

Supplementary Table 1A: Patient characteristics
| BRx140 | |
| --- | --- |
| Age at Primary Diagnosis | 58 |
| Age at Metastatic Diagnosis | 67 |
| De Novo Metastasis | No |
| Number of Prior Endocrine Therapies | 1 |
| Number of Prior Chemotherapies | 0 |
| Received Prior CDK 4/6 Therapies | No |
| Prior Adjuvant Aromatase Inhibitor | Yes |
| BRx50 | |
| --- | --- |
| Age at Primary Diagnosis | 32 |
| Age at Metastatic Diagnosis | 39 |
| De Novo Metastasis | No |
| Number of Prior Endocrine Therapies | 1 |
| Number of Prior Chemotherapies | 2 |
| Received Prior CDK 4/6 Therapies | No |
| Prior Adjuvant Aromatase Inhibitor | No |

## Slide 4
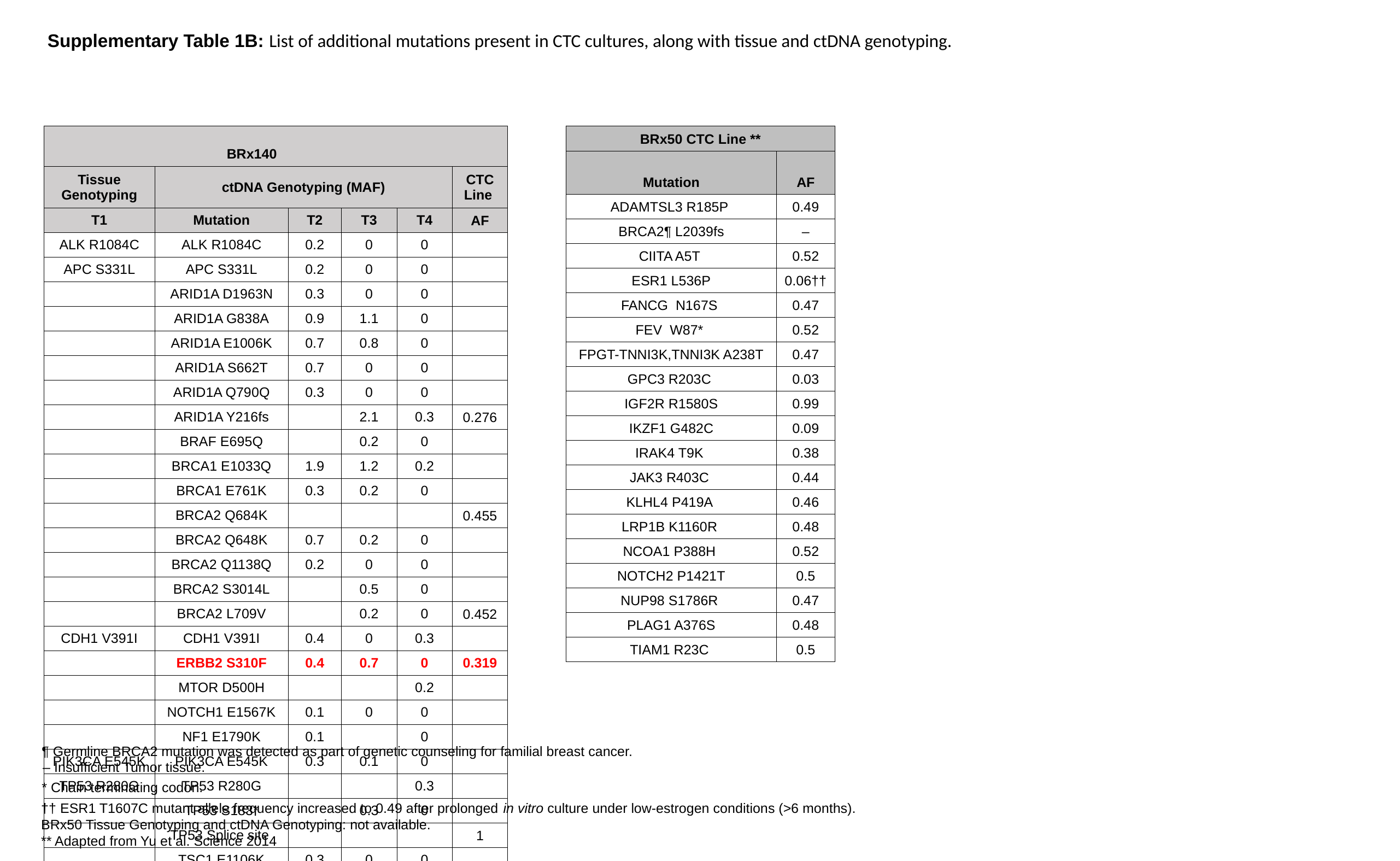

Supplementary Table 1B: List of additional mutations present in CTC cultures, along with tissue and ctDNA genotyping.
| BRx140 | | | | | |
| --- | --- | --- | --- | --- | --- |
| Tissue Genotyping | ctDNA Genotyping (MAF) | | | | CTC Line |
| T1 | Mutation | T2 | T3 | T4 | AF |
| ALK R1084C | ALK R1084C | 0.2 | 0 | 0 | |
| APC S331L | APC S331L | 0.2 | 0 | 0 | |
| | ARID1A D1963N | 0.3 | 0 | 0 | |
| | ARID1A G838A | 0.9 | 1.1 | 0 | |
| | ARID1A E1006K | 0.7 | 0.8 | 0 | |
| | ARID1A S662T | 0.7 | 0 | 0 | |
| | ARID1A Q790Q | 0.3 | 0 | 0 | |
| | ARID1A Y216fs | | 2.1 | 0.3 | 0.276 |
| | BRAF E695Q | | 0.2 | 0 | |
| | BRCA1 E1033Q | 1.9 | 1.2 | 0.2 | |
| | BRCA1 E761K | 0.3 | 0.2 | 0 | |
| | BRCA2 Q684K | | | | 0.455 |
| | BRCA2 Q648K | 0.7 | 0.2 | 0 | |
| | BRCA2 Q1138Q | 0.2 | 0 | 0 | |
| | BRCA2 S3014L | | 0.5 | 0 | |
| | BRCA2 L709V | | 0.2 | 0 | 0.452 |
| CDH1 V391I | CDH1 V391I | 0.4 | 0 | 0.3 | |
| | ERBB2 S310F | 0.4 | 0.7 | 0 | 0.319 |
| | MTOR D500H | | | 0.2 | |
| | NOTCH1 E1567K | 0.1 | 0 | 0 | |
| | NF1 E1790K | 0.1 | | 0 | |
| PIK3CA E545K | PIK3CA E545K | 0.3 | 0.1 | 0 | |
| TP53 R280G | TP53 R280G | | | 0.3 | |
| | TP53 S183\* | | 0.3 | 0 | |
| | TP53 Splice site | | | | 1 |
| | TSC1 E1106K | 0.3 | 0 | 0 | |
| BRx50 CTC Line \*\* | |
| --- | --- |
| Mutation | AF |
| ADAMTSL3 R185P | 0.49 |
| BRCA2¶ L2039fs | – |
| CIITA A5T | 0.52 |
| ESR1 L536P | 0.06†† |
| FANCG N167S | 0.47 |
| FEV W87\* | 0.52 |
| FPGT-TNNI3K,TNNI3K A238T | 0.47 |
| GPC3 R203C | 0.03 |
| IGF2R R1580S | 0.99 |
| IKZF1 G482C | 0.09 |
| IRAK4 T9K | 0.38 |
| JAK3 R403C | 0.44 |
| KLHL4 P419A | 0.46 |
| LRP1B K1160R | 0.48 |
| NCOA1 P388H | 0.52 |
| NOTCH2 P1421T | 0.5 |
| NUP98 S1786R | 0.47 |
| PLAG1 A376S | 0.48 |
| TIAM1 R23C | 0.5 |
¶ Germline BRCA2 mutation was detected as part of genetic counseling for familial breast cancer.
– Insufficient Tumor tissue.
* Chain terminating codon.
†† ESR1 T1607C mutant allele frequency increased to 0.49 after prolonged in vitro culture under low-estrogen conditions (>6 months).
BRx50 Tissue Genotyping and ctDNA Genotyping: not available.
** Adapted from Yu et al. Science 2014
